# Microglia–Neutrophil Interactions Drive Dry AMD-like Pathology in a Mouse Model

**DOI:** 10.3390/cells11223535

**Published:** 2022-11-09

**Authors:** Maeve Boyce, Ying Xin, Olivia Chowdhury, Peng Shang, Haitao Liu, Victoria Koontz, Anastasia Strizhakova, Mihir Nemani, Stacey Hose, J. Samuel Zigler, Matthew Campbell, Debasish Sinha, James T. Handa, Kai Kaarniranta, Jiang Qian, Sayan Ghosh

**Affiliations:** 1 Department of Ophthalmology, School of Medicine, University of Pittsburgh, Pittsburgh, PA 15260, USA; 2 Smurfit Institute of Genetics, Trinity College, D02 PN40 Dublin, Ireland; 3 Department of Ophthalmology, The Wilmer Eye Institute, The Johns Hopkins School of Medicine, Baltimore, MD 21205, USA; 4 Department of Ophthalmology, Kuopio University Hospital, University of Eastern Finland, 70211 Kuopio, Finland

**Keywords:** age-related macular degeneration, retinal pigmented epithelial cells, microglia, neutrophils, chronic inflammation, Akt2, LR-loop, CD14, integrin β1, integrin α4

## Abstract

In dry age-related macular degeneration (AMD), inflammation plays a key role in disease pathogenesis. Innate immune cells such as microglia and neutrophils infiltrate the sub-retinal space (SRS) to induce chronic inflammation and AMD progression. But a major gap in our understanding is how these cells interact with each other in AMD. Here, we report a novel concept of how dynamic interactions between microglia and neutrophils contribute to AMD pathology. Using well-characterized genetically engineered mouse models as tools, we show that in the diseased state, retinal pigmented epithelial (RPE) cells trigger pro-inflammatory (M1) transition in microglia with diminished expression of the homeostatic marker, CX3CR1. Activated microglia localize to the SRS and regulate local neutrophil function, triggering their activation and thereby inducing early RPE changes. Ligand receptor (LR)-loop analysis and cell culture studies revealed that M1 microglia also induce the expression of neutrophil adhesion mediators (integrin β1/α4) through their interaction with CD14 on microglia. Furthermore, microglia-induced neutrophil activation and subsequent neutrophil-mediated RPE alterations were mitigated by inhibiting Akt2 in microglia. These results suggest that the Akt2 pathway in microglia drives M1 microglia-mediated neutrophil activation, thereby triggering early RPE degeneration and is a novel therapeutic target for early AMD, a stage without treatment options.

## 1. Introduction

Inflammation plays an important role in maintaining tissue homeostasis [1]. However, dysregulation of the inflammatory response is associated with tissue damage and the onset of several aging diseases, including age-related macular degeneration (AMD) [2,3,4], the leading cause of blindness in the elderly [5]. Dry AMD is the major form of the disease and unfortunately to date, is without effective treatment [6]. The retinal pigmented epithelial (RPE) cells, which are the first cells to be affected in dry AMD [7], are subjected to low-grade protective (para) inflammatory changes with aging thereby activating resident immune cells such as microglia [8,9,10], which act to maintain retinal homeostasis [10]. However, environmental risk factors and genetic predispositions can initiate an unregulated inflammatory response during aging, thereby triggering detrimental (chronic) inflammation in the retina [2,3,4]. Chronic inflammation is associated with a prolonged heightened immune response, breakdown of the blood–retinal barrier, activation of complement factors, and migration of microglia, monocytes, and neutrophils to the sub-retinal space (SRS)—a region now thought to be critical for the para to chronic inflammatory transition in AMD pathogenesis [11,12,13,14,15,16,17].

In the SRS, the RPE cells play a key role in the activation and infiltration of the innate immune cells including microglia, neutrophils, and monocytes, key immune cell types that have previously been shown to be important for AMD progression [12,14,17]. Microglia can have both protective and detrimental functions in the retina during retinal homeostasis as well as degeneration, which can be governed by specific cues from the RPE [11,12,15,18]. In addition, we have shown that RPE cells trigger neutrophil activation and infiltration into the retina [17] of a mouse model of dry AMD which lacks the *Cryba1* gene (encodes βA3/A1-crystallin) specifically in the RPE (conditional knockout; cKO) [19,20] and in human dry AMD patients [16,17]. Moreover, neutrophil homing into the retina is associated with retinal degeneration as seen in AMD [17]. A major gap in our understanding thus far is how these immune cells interact with one another in the SRS during AMD pathogenesis to potentiate chronic inflammation and retinal degeneration.

We speculate that during AMD pathogenesis, activated microglia interact with infiltrating neutrophils in the SRS to aggravate retinal inflammation and trigger retinal degeneration. Understanding the molecular pathways that regulate this dynamic interaction between a tissue resident immune cell (microglia) and a peripheral immune cell (neutrophil) and the impact these interactions have on the retina during disease progression will stimulate future drug discoveries for this debilitating disease.

Herein, we demonstrate that this dynamic interaction between microglia and neutrophils is important in the AMD pathogenesis in mouse models. We show that RPE-derived soluble factors trigger microglial activation and pro-inflammatory (M1) transition in the diseased state due to Akt2 activation in microglia. M1 microglia activate neutrophils as suggested by neutrophil extracellular traps (NET) formation, along with increased lipocalin-2 (LCN-2) and myeloperoxidase (MPO) levels. Activated neutrophils, in turn, induce early RPE morphological alterations. Further, the pro-inflammatory microglia also upregulate adhesion factors such as integrins β1 and α4 on neutrophils, which are critical for transmigration into the tissue, via activation of CD14 (microglia)/integrin β1 and α4 (neutrophils) interactions between the two immune cells. Intriguingly, targeting Akt2 in microglia with a specific inhibitor reduced the pro-inflammatory transition in microglial cells and subsequently reduced neutrophil activation in vitro, as well as neutrophil mediated RPE alterations in vivo, which suggests that this pathway is a novel therapeutic target for early, dry AMD.

## 2. Materials and Methods

### 2.1. Antibodies

Antibodies for flow cytometry which were purchased from BD Bioscience, Franklin Lakes, NJ, USA are PE/Cy7-tagged CD45 (Cat# 103114), BV421-tagged Ly6C (Cat# 128016), APC-tagged Ly6G (Cat# 560599), Alexa fluor 700-tagged CD11b (Cat# 557960). Other antibodies used are PerCP-Cy5.5-tagged CX3CR1 (Biolegend, San Diego, CA, USA; Cat# 149009), FITC-tagged CD14 (Thermo Fisher, Waltham, MA, USA; Cat# 11-0141-81), anti-AKT2 (Cell Signaling Technologies, Danvers, MA, USA; Cat# 2964S), Alexa fluor 488-tagged integrin β1 (Santa Cruz Biotechnology, Dallas, TX, USA; Cat# sc-374429 AF488), integrin α4 (Cell Signaling Technologies, USA; 8440S), and Actin (Cell Signaling Technologies, USA; Cat# 4970S).

### 2.2. Animals

All animal studies were conducted in accordance with the Guide for the Care and Use of Animals (National Academy Press) and were approved by the University of Pittsburgh Animal Care and Use Committee (Protocol # 20108281). Both male and female mice were used for this study [17,21]. RPE-specific *Akt2* KI were generated as described previously [22]. The RPE-specific *Akt2* KI mice were generated by Cyagen. Briefly, the T2A sequence followed by Akt2 coding sequence (CDS) was inserted between the last exon and the 3′ untranslated region (3′UTR) of the mouse *Best1* gene, which in the eye is specifically expressed in the RPE. The Neo cassette flanked with self-deletion anchors (SDA) was inserted in the intron area between exons 11 and 12 of the mouse *Best1* for germ cell deletion of the gene. βA3/A1-crystallin conditional (*Cryba1* cKO) [17,19,20] and complete knockout (*Cryba1* KO) [16,21] mice were also generated and maintained as previously described [16,17,19,20,21,22]. Nonobese diabetic/severe combined immunodeficiency (NOD-SCID) mice (NOD.CB17-Prkdescid/J; 5 weeks old) were purchased from The Jackson Laboratory, USA. All mice used in this study were RD8 negative.

### 2.3. RPE Explant Culture

RPE explants from 10 month old WT, *Cryba1* KO and *Akt2* KI mice were cultured as explained previously [23]. Briefly, fresh eyes were enucleated, and the anterior segment was removed. The neural retina was carefully removed and then the posterior eye cup was dissected into four petals. The resulting RPE-choroid-sclera (RCS) complexes were flattened onto polyvinylidene difluoride (PVDF) membranes with the RPE cells facing up and cultured as previously described [17]. The RPE spent medium (RPESM) was harvested after 24 h by carefully removing the media for further experiments [17].

### 2.4. Microglia Culture

Mouse microglial cells were purchased from ScienCell, Carlsbad, CA, USA (Cat# M1900-57) and were cultured according to the manufacturer’s protocol. For co-culture experiments only, microglia were plated (1 × 10^5^ cells/mL) on the underside of the insert of transwell plates (Cat# COR-3460, Corning, Glendale, AZ, USA). At 80% confluency, the microglial cells were exposed to RPESM from WT, *Cryba1* KO, and *Akt2* RPE explant cultures at a dilution of 1:1 (RPESM: microglia media) for 24 h. Akt2 inhibitor (CCT128930: Selleckchem, Houston, TX, USA; Cat# S2635) treatment at a dose of 5 nM or vehicle only (2.5% dimethyl sulfoxide; DMSO in phosphate-buffered saline; PBS) was given at the time of RPESM exposure for 24 h. CD14 neutralization was performed 2 h before the start of the neutrophil co-culture using a neutralizing antibody against CD14 (Thermo Fisher, USA; Cat# 14-0149-82) at a dose of 1 µg/mL and was treated until end of the experimental duration.

### 2.5. Neutrophil Culture

Neutrophils from WT mice were isolated as previously described [17]. Briefly, bone marrow cells were isolated from femurs and tibias and purified over a Percoll discontinuous density gradient. After isolation, neutrophils were resuspended at a density of 10 × 10^6^ cells/mL in Ca^2+^ and Mg^2+^ free Hank’s balanced salt solution (HBSS), supplemented with 20 mM HEPES and then co-cultured with microglial cells for 4 h at 3 × 10^4^ cells/mL (neutrophil: microglia = 1:10), as explained previously with minor modifications [24]. Briefly, transwell plates with microglia (from different experimental groups) were exposed to WT neutrophils on the apical surface for 4 h. Neutrophil viability was measured by trypan blue dye exclusion after 4 h of incubation.

### 2.6. Immunophenotyping of SRS

The SRS (including RPE-choroid) was dissected following perfusion to remove immune cells from choroid blood vessels as described previously [23]. The tissues were then digested with 0.05% collagenase D (Roche, Switzerland, Cat# 11088858001) at 37 °C for 30 min, teased out with a blunt end forceps and pipetted 10 times to release the cells. The cell suspension was then passed through a 70 µm cell strainer, and centrifuged at 1300× *g* at 4 °C for 20 min. The entire pellet was first blocked with Fc blocker and 2% each of rat and mouse serum in 5% bovine serum albumin (BSA) in PBS for 30 min at room temperature. Dead cells were then removed from the cell suspension using the cell debris removal solution (Miltenyi Biotec, Gaithersburg, MD, USA, Cat# 130-109-398). Flow cytometry was used to assess the % neutrophils in the parent cell population: CD45^high^CD11b^+^ cells (see Supplementary Figure S1) and microglia, after staining with anti-Ly6G, Ly6C, CD11b, and CD45, CX3CR1 antibodies at a concentration of 1 µg/mL for 90 min at room temperature, as previously described [17].

### 2.7. Evaluation of Cell Surface Receptor Expression

Freshly cultured bone marrow-derived neutrophils from WT mice co-cultured with microglia from the different experimental conditions (as outlined above) were incubated with Alexa fluor 488-tagged Integrin β1 (Santa Cruz Biotechnology, USA) antibodies or Alexa fluor 568-tagged Integrin α4 (Cell Signaling Technology, USA) at a concentration of 1 µg/mL in PBS containing 1% BSA for 1 h [17]. The cell surface expression of integrin β1 (FITC-A Mean fluorescence) and integrin α4 (PE-A Mean fluorescence) was evaluated among these cells using BD Fortessa flow cytometer (BD Biosciences, Franklin Lakes, NJ, USA). Results were analyzed as described previously using FlowJo software [17].

### 2.8. Cytometry Bead Array (CBA)

Microglia spent medium and RPE lysates from different the experimental groups were used to ascertain the levels of cytokines and chemokines using the Legendplex mouse proinflammatory chemokine panel (Biolegend, USA; Cat# 740451) and Legendplex mouse inflammation panel (Biolegend, USA; Cat# 740446). The experiments were performed, and the results were analyzed using the manufacturer’s protocols.

### 2.9. Single Cell (sc) RNA Sequencing and Bioinformatics

The SRS (including RPE-choroid) was dissected following perfusion to remove immune cells from choroid blood vessels as described previously [23] from 15 month old *Cryba1*-floxed and cKO mice. The single cell suspension was subjected to scRNAseq as a paid service from the Genomics Research Core of University of Pittsburgh to identify the RNA expression profile of different cells. Bioinformatics analysis was performed by creating Seurat objects for each sample by the function “CreateSeuratObject” in Seurat package and then filtering out cells with nFeature_RNA > 8000, nFeature_RNA < 250, nUMI > 40,000, nUMI < 500, log10 (GenesPerUMI) < 0.8, or with a mitochondrial rate > 20%. Scrublet was used to remove predicted doublets with default parameters [23]. Then genes detected in fewer than 5 cells were also removed. As a result, the expression of 17,626 genes in 10,134 cells were used for downstream analysis. After clustering the cells, cell type identities were assigned based on the top marker genes of each cluster as well as visualizing the expression of specific marker genes of candidate cell types [23]. Average expression values of the genes in each cell type and each sample were then calculated by the function “AverageExpression” in Seurat package. Differential expression analysis was performed on each cell type of interest between *Cryba1*-floxed and cKO samples by the function FindMarkers with test.use = “wilcox” in Seurat package [23].

For the analysis of ligand–receptor interactions between cells, the default built-in ligand-receptor interaction database, intracellular signaling database and gene regulatory networks in the package LRLoop were used [25]. The ligand–receptor pairs and intracellular signaling interactions with a detection rate less than 2.5% in the corresponding cell types were removed [25]. Analysis of the remaining candidate ligand–receptor pairs between each pair of cell types of interest in each sample was performed using the LRLoop package with its standard pipeline and default parameters [23].

### 2.10. Subretinal Injection

NOD-SCID mice (Males, 5 weeks-old) were used for the study [17,23]. Mice were anaesthetized by intraperitoneal injection of a mixture of ketamine (Covetrus, Dublin, OH, USA; Cat# 071069) at a dose of 50 mg/kg body weight and xylazine (Sigma Aldrich, Cat# X1251) at a dose of 10 mg/kg body weight, and sub-retinal injections [17,23] of neutrophils (1 µL; at a concentration of 5 × 10^6^ cells/mL) from different experimental groups (co-cultured with microglia exposed to RPESM from WT, *Cryba1* KO and *Akt2* KI RPE explant cultures) were given to each mouse. Three days after the injections, the NOD-SCID mice were euthanized, and the eyes were enucleated for RPE flat mount preparations [17,21].

### 2.11. RPE Flatmount and Phalloidin Staining

Eyes were freshly enucleated and then fixed in 2% paraformaldehyde (PFA) for 10 min followed by the removal of the anterior segment (cornea, lens, and attached iris pigmented epithelium). The resulting posterior eyecups were fixed in 2% PFA for 1 h at room temperature for RPE flat mount preparation [17,21]. Tissues were removed after the eyecup was quartered into a petaloid structure and the neural retina was carefully removed. The resulting eyecup was further cut radially into eight pieces from the optic nerve head to the periphery. Immunostaining on RPE flatmounts was performed by using Alexafluor 488-conjugated phalloidin (1:1000) (Thermo Fisher, USA; Cat# A12379) with 1 µg/mL DAPI (Thermo Fisher, USA; Cat# 62248) and incubated at room temperature for 1 h [17,21]. The flatmounts were washed six times with 1× tris-buffered saline (TBS), mounted on cover slips with DAKO mounting agent (Agilent, Santa Clara, CA, USA, Cat# S3023), and then visualized under a confocal microscope (Zeiss LSM710, Oberkochen, Germany) to assess RPE morphological changes [17,21].

### 2.12. Retinal Cryosections and Immunostaining

Whole eyes from freshly dissected mice were enucleated and fixed in 2% paraformaldehyde (PFA) for 10 min and then the anterior parts were removed. The posterior eyecups were again fixed in 2% PFA for 1 h at room temperature. Immunofluorescence was performed on frozen sections from the posterior eyecups as explained previously [16,21]. The sections were incubated with phosphate-buffered saline, containing 5% normal donkey or goat serum, for 30 min and then incubated overnight at 4 °C with primary antibodies for Iba1 (Wako, Japan; Cat# 019-19741) or Ly6G (Biorbyt, St. Louis, MO, USA; Cat# orb322983) diluted to 1:100. The sections were washed with TBS and then incubated at room temperature with respective secondary antibodies with 1 µg/mL DAPI (Thermo Fisher, USA; Cat# 62248) and +/− Alexafluor 488-conjugated phalloidin (1:1000) (Thermo Fisher, USA; Cat# A12379). Sections were again washed with TBS and then mounted using DAKO mounting agent (Agilent, USA, Cat# S3023). Images were acquired by a Zeiss LSM 710 confocal workstation.

### 2.13. Western Blotting

Western blotting was performed to evaluate the expression of Akt2 in microglia lysates using previously published methods [17,21]. The microglia cells were lysed with 1× RIPA buffer (EMD Millipore, Burlington, MA, USA; Cat# 20–188) containing 0.1% of protease inhibitor cocktail (Sigma Aldrich, Burlington, MA, USA, I3786) and 0.1% phosphatase inhibitor cocktail (Sigma Aldrich, USA; Cat# P0044-5ML). Protein quantification was then performed on the lysates using the Pierce BCA Protein Assay Kit (Thermo Fisher, USA; Cat# 23225). The RIPA protein samples were mixed with 4× protein sample buffer (Life Technologies, Carlsbad, CA, USA; Cat# NP0007) containing 5% 2-mercaptoethanol (Sigma Aldrich, USA; Cat# M6256) and heated at 95 °C for 10 min to denature [17,21]. Samples were subjected to SDS-PAGE using the 4–12% Bis-Tris Nu-PAGE gel (Invitrogen, Waltham, MA, USA; Cat# NP0323BOX) and MES buffer (Invitrogen, USA; Cat# NP0002). Proteins were transferred to nitrocellulose membranes (Invitrogen, USA; Cat# IB23001), which were then blocked in 5% blocking grade skim milk (Bio-Rad, USA; Cat# 170–6404). The membranes were incubated with the appropriate primary antibody overnight followed by horseradish peroxidase-conjugated anti-rabbit (KPL, Gaithersburg, MD, USA; Cat# 074–1506) secondary antibody for 2 h at RT [17,21]. The membranes were developed using a chemiluminescence technique (GE Healthcare, Chicago, IL, USA; Cat# RPN2209) and then imaged using the Azure imaging system (Azure Biosystems, Dublin, CA, USA). Densitometry was performed to estimate the protein expression relative to the loading control (Actin) using ImageJ software (National Institutes of Health, Bethesda, MD, USA) [17,21].

### 2.14. Enzyme-Linked Immunosorbent Assay (ELISA)

Neutrophils were lysed in 100 µL of complete extraction buffer (Abcam, Boston, MA, USA, Cat# ab193970) and the levels of LCN-2 (Invitrogen, USA; Cat# EMLCN2) and MPO (Invitrogen, USA; Cat# EMMPO) were evaluated using the manufacturer’s protocol.

### 2.15. Statistical Analysis

Graphs were generated using Microsoft Excel and GraphPad 8.0 software [17,21,23]. Statistical analyses were done using one-way ANOVA followed by Tukey post hoc test to measure the differences between groups [17,21,23]. The significance was set at *p* < 0.05. Each biological replicate had at least three technical replicates. Results are presented as mean ± standard deviation (SD) [17,21,23]. 

## 3. Results

### 3.1. Microglia and Neutrophils Infiltrate the SRS in a Mouse Model upon Activation of Retinal Inflammation

**Figure 1:**
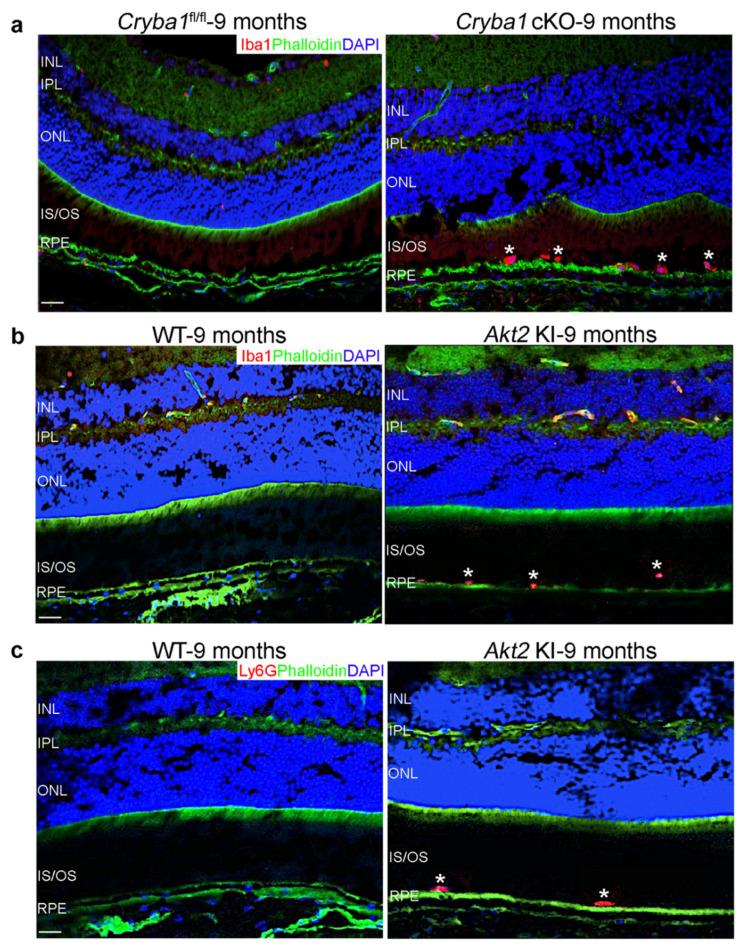
Infiltration of microglia and neutrophils into the SRS of aged *Cryba1* cKO and *Akt2* KI mice. (**a**) Immunofluorescence studies showing accumulation of Iba1-positive microglial cells (Iba1: Red, asterisks) in the SRS of retina sections from 9 month old *Cryba1* cKO mice, but not in age-matched *Cryba1*^fl/fl^ retina sections. Scale bar = 100 µm. (**b**,**c**) Retina sections from 9 month old *Akt2* KI mice showing subretinal infiltration of microglia (Iba1: Red, asterisks in (**b**), and neutrophils (Ly6G: Red, asterisks in (**c**). Green: Phalloidin, Blue: DAPI; nucleus. Scale bar = 100 µm. n = 3.

We have previously shown that in the *Cryba1* cKO mouse model with a dry AMD-like phenotype [19], microglia are activated [20], and neutrophils infiltrate the SRS with age [17]. To assess the spatiotemporal localization of the activated microglia in aged *Cryba1* cKO mice, we performed immunofluorescence studies on retinal cryo-sections and found that microglia (Iba1-positive cells) home into the SRS of aged (9 months old; the age when the AMD-like phenotype is observed in this mouse model) *Cryba1* cKO retina, but not in age-matched floxed (*Cryba1*^fl/fl^) controls (Figure 1a). We also found accumulation of microglia (Iba1-positive cells) and neutrophils (Ly6G-positive cells) (Figure 1b,c) in the SRS of aged (9 months old) *Akt2* KI mice, a genetically engineered mouse model with constitutive activation of Akt2 specifically in the RPE cells [22]. Akt2 is a kinase which has been previously shown to be critical for inflammation activation [26,27] and is upregulated in RPE cells from human AMD patients as well as in the *Cryba1* cKO mouse model [16,17]. Since Iba1 is expressed in both microglia and macrophages/monocytes [11,15], these Iba1+ cells could be either microglia or macrophage lineage cells. In addition to microglia, macrophages/monocytes are also known to infiltrate the SRS in animal models and human AMD patients [11,13,15]. 

**Figure 2:**
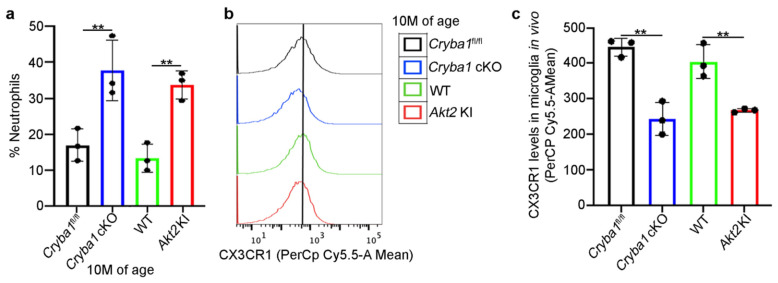
Accumulation of neutrophils and microglia in the SRS of mouse models. (**a**) Cells isolated from the SRS of 10 month old *Cryba1* cKO (when the AMD-like phenotype is first observed in the *Cryba1* cKO mouse model) and *Akt2* KI mice showed increase in neutrophils (% neutrophils), compared to age matched floxed and WT mice. (**b**) Flow cytometric fluorescence plot and (**c**) quantification of microglia homeostatic marker, CX3CR1 (PerCpCy5.5-A mean fluorescence) showing diminished expression of the protein in microglia (CD45^Low^CD11b^+^Ly6C^−^Ly6G^−^) from the SRS of 10-month-old *Cryba1* cKO and *Akt2* KI mice, compared to controls. n = 3. ** *p* < 0.01.

To substantiate the sub-retinal accumulation of neutrophils and the presence/activation of microglia or macrophages in these mouse models, we performed immunophenotyping for multiple cell surface markers to differentiate the various immune cells in the sub-retinal tissue (including RPE/choroid) by flow cytometry [17], finding significant elevation in the number of neutrophils in 10 month old *Cryba1* cKO and *Akt2* KI mice (Figure 2a and Figure S1). Interestingly, we also found a decline in the expression of the homeostatic marker, CX3CR1 among the microglial cells (Figure 2b,c and Figure S1). However, no significant increase was observed in the number of monocytes or macrophages in the *Cryba1* cKO and *Akt2* KI mice, compared to controls (Figure 2b,c and Figure S1). CX3CR1 has been previously demonstrated to be associated with AMD, both in animal models and human AMD patients [11,15]. Loss of function of CX3CR1 has been shown to be critical in peripheral immune cell infiltration into the retina as well as in the induction of chronic inflammation and AMD-like pathology in mouse models [11,15]. It is likely that in our mouse models, the RPE-mediated activation of the proinflammatory niche in the SRS is the cause of immune cell infiltration.

### 3.2. RPE Drives Microglia Activation through the Akt2 Signaling Pathway

**Figure 3:**
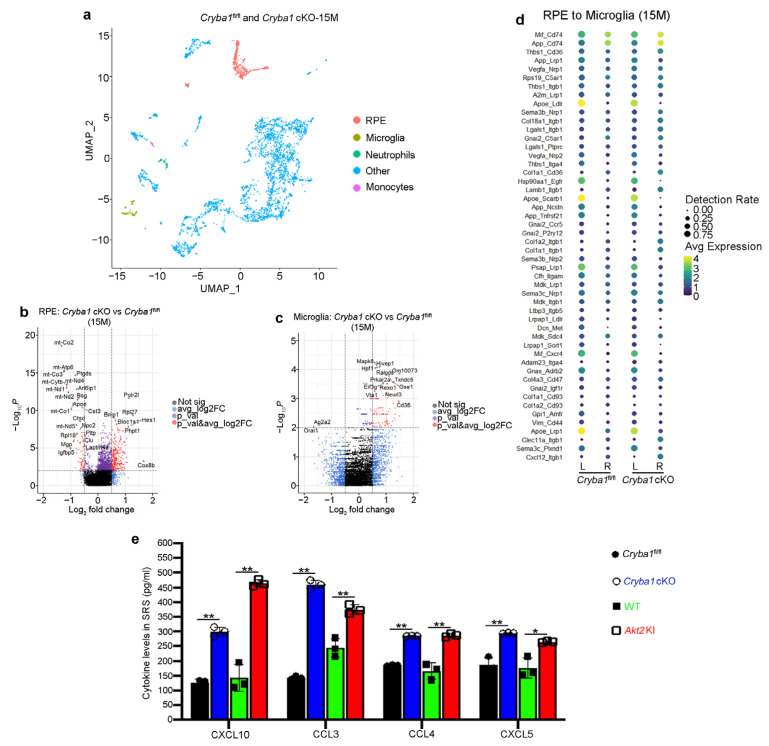
*Cryba1* cKO RPE cells interact with microglial cells in the disease state. (**a**) UMAP plot generated from integrating cells from all samples (15 month old *Cryba1*-floxed and cKO mice) showing, RPE cluster (red) and other cell clusters (blue), along with the clusters of different immune cells known to be critical in AMD pathogenesis: microglia (brown), neutrophils (green), and monocytes (magenta). (**b**,**c**) Volcano plots showing differential gene expression [log2Fold change (FC)] in (**b**) RPE and (**c**) microglia between *Cryba1* cKO and floxed controls at 15 months of age. (**d**) Ligand (L): receptor (R) pairs analysis showing expression levels (average expression and detection rate of the gene) of the indicated ligands (in RPE) and receptors (in microglia) in *Cryba1* cKO and floxed mice. Top 50 RPE-microglia interactive partners are represented. (**e**) Cytometry bead array analysis revealed elevated levels of the pro-inflammatory chemokines CXCL10, CCL3, CCL4, and CXCL5 in RPE lysates from 10 month old *Cryba1* cKO and *Akt2* KI, compared to age-matched controls. n = 3. * *p* < 0.05, ** *p* < 0.01.

The RPE is the first cells that get affected in dry AMD [7]. It is known that diseased RPE cells trigger infiltration of the major retinal resident immune cells, microglia, into the SRS, thereby exacerbating the inflammatory processes as evident from elevated levels of pro-inflammatory cytokines and homing of peripheral immune cells such as neutrophils, monocytes, and mast cells into the retina [8,9,10,11,15,28]. This heightened inflammatory state subsequently contributes to RPE loss and photoreceptor degeneration in AMD [2,3,4]. To understand the underlying mechanisms by which the RPE cells could induce microglia activation during AMD pathogenesis, we performed scRNAseq analysis on cells isolated from the SRS (including RPE and choroid) of aged (15 months old) *Cryba1*^fl/fl^ and *Cryba1* cKO mice [23] and identified 35 different cell clusters including the RPE [23] along with immune cell (microglia, neutrophils, monocytes) populations (Supplementary Figure S2), that have been previously documented to be important in AMD pathogenesis [10,11,12,13,14,15,16,17,18]. Further, in-depth analysis revealed several differentially expressed genes in the RPE and microglial cells (Figure 3b,c). To pinpoint the signaling molecules associated with RPE–microglia interaction in the disease state of this mouse model, we performed a novel ligand-receptor (LR) loop bioinformatic analysis [25] that showed several interactive partners linking RPE (Ligand; L) and microglia (Receptor; R) in 15-month-old *Cryba1*^fl/fl^ and *Cryba1* cKO (Figure 3d). The LR loop interaction between RPE and microglia revealed a notable increase in the interactions between cognate ligands from RPE cells (such as CXCL12 and Col4a3) and their respective cell surface receptors such as Itgb1 and CD47 on microglia. We also observed a decrease in CD11b on microglia in aged *Cryba1* cKO (Figure 3d), a change previously shown to be important for pathogenic accumulation of these immune cells in the SRS leading to chronic inflammation and retinal degeneration [11,14]. We have also previously shown that RPE cells from the *Cryba1* cKO mice express high levels of several pro-inflammatory cytokines and chemokines [17]. To further assess the levels of pro-inflammatory chemokines in the RPE cells, we performed a cytometry bead array (CBA) on RPE lysates from 10-month-old WT, *Cryba1* cKO and *Akt2* KI mice. Our results showed significant upregulation of CXCL10, CCL3, CCL4, and CXCL5 in *Cryba1* cKO and *Akt2* KI RPE cells, relative to controls (Figure 3e). Chemokines, such as CXCL10 and CCL4 have been previously documented to be essential for microglial activation [29,30], whereas CXCL10, CCL3, and CXCL5 have been associated with AMD pathogenesis [31,32]. It is likely that these pro-inflammatory mediators are secreted by the RPE, but infiltrating immune cells at the SRS could also contribute to the levels of these cytokines as explained previously [12,14,16,17], and such pro-inflammatory contributions from different cell types at the SRS might be responsible for the age-dependent activation and migration of microglia and other immune cells into the SRS in these mouse models.

**Figure 4:**
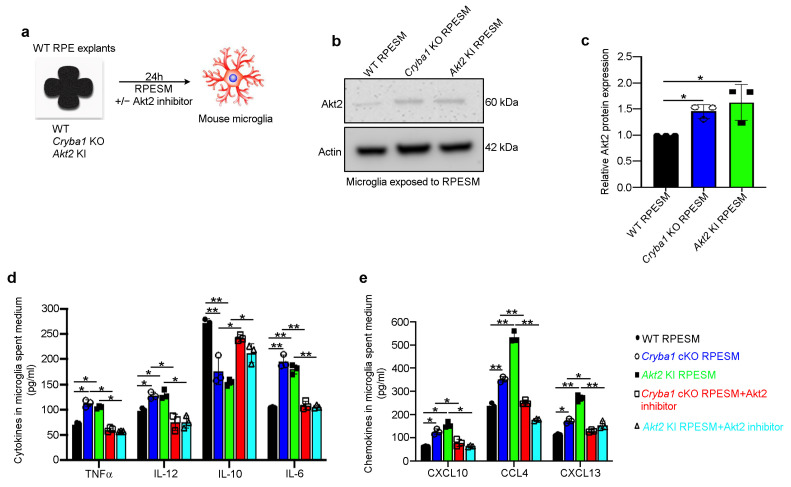
RPE-mediated microglial activation is regulated by Akt2. (**a**) Schematic showing experimental design; RPE spent medium (RPESM) harvested from 24 h RPE explant cultures from aged WT, *Cryba1* KO and *Akt2* KI mice was used to culture mouse microglia for 24 h with or without 5 nM Akt2 inhibitor. (**b**) Western blot and (**c**) densitometry showing that Akt2 is upregulated in microglial cells following exposure to RPESM from *Cryba1* KO and *Akt2* KI RPE explant cultures, relative to WT RPESM-exposed microglia. (**d**) Cytometry bead array analysis revealed significant upregulation of M1 mediators TNFα, IL-12, and IL-6 and downregulation of the M2 mediator IL-10 in MSM from *Cryba1* KO and *Akt2* KI RPESM-exposed microglia relative to WT RPESM-exposed cells. This trend indicates a transition to the pro-inflammatory M1 state in these microglia. (**e**) Chemokines such as CXCL10, CCL4, and CXCL13 were also increased in the spent medium from *Cryba1* KO and *Akt2* KI RPESM-exposed microglia, compared to controls. Surprisingly, adding Akt2 inhibitor to the microglia culture medium rescued the levels of these pro-inflammatory mediators to near control values (**d**,**e**). n = 3. * *p* < 0.05, ** *p* < 0.01.

We have previously used *Cryba1* KO RPE explants for harvesting RPESM for in vitro experiments since in *Cryba1* KO mice, the *Cryba1* gene that encodes the βA3/A1-crystallin protein, is completely absent in all RPE cells, whereas the *Cryba1* cKO mice retain about 15% of the normal complement of βA3/A1-crystallin protein due to the mosaic expression of *Best1*-Cre, which was used to generate *Cryba1* cKO animals [23]. In addition, *Cryba1* KO mice develop a heightened inflammatory response with age and an early AMD-like phenotype [16,21]. To confirm that pro-inflammatory mediators secreted by RPE could trigger microglial activation, RPESM harvested from RPE explant cultures (24 h culture) from aged WT, *Cryba1* KO (complete knockout), and *Akt2* KI mice was added to cultures of mouse microglia. After 24 h (Figure 4a), Western blot analysis showed significant upregulation of Akt2, a known regulator/activator of pro-inflammatory (M1) microglia [33] in lysates from *Cryba1* KO and *Akt2* KI RPESM-exposed microglia, compared to those from WT RPESM-exposed microglia (Figure 4b,c). Further, CBA analysis from microglia spent medium (MSM) revealed significantly increased levels of pro-inflammatory (M1) mediators [34] such as TNFα, IL-12, and IL-6 and a decrease in the level of the M2 mediator IL-10 [34], in the MSM of *Cryba1* KO and *Akt2* KI RPESM-exposed microglial cells (Figure 4d). Compared to WT RPESM treated microglia, cells exposed to *Cryba1* KO and *Akt2* KI RPESM showed significantly increased levels of the chemokines CXCL10, CCL4, CXCL13 (Figure 4e), which are known to be activated during M1 transition in macrophage-lineage cells, e.g., microglia [29,35]. Interestingly, inhibition of Akt2 in microglia using a specific Akt2 inhibitor, CCT128930 [17] (5 nM) for 24 h significantly decreased pro-inflammatory cytokines and chemokines levels released from microglia even following exposure to *Cryba1* KO or *Akt2* KI RPESM (Figure 4d,e). These results demonstrate that targeting Akt2 can reverse microglia activation in vitro.

### 3.3. Microglia and Neutrophils Interact in the SRS of a Mouse Model of Dry AMD

**Figure 5:**
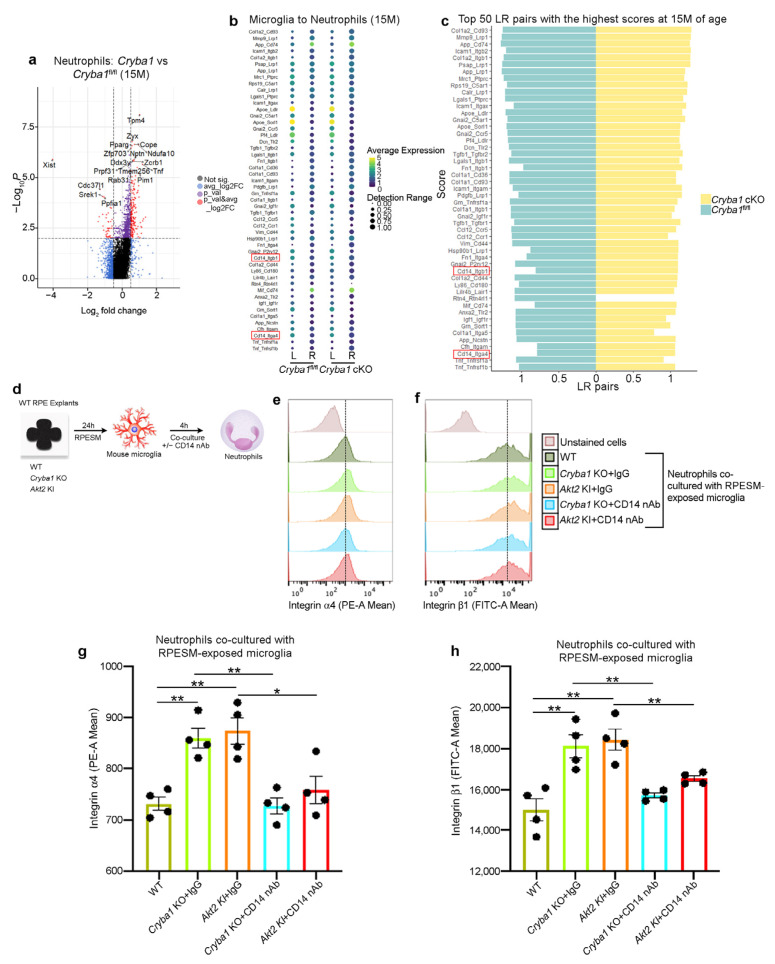
CD14 on microglia regulates expression of adhesion proteins on neutrophils. (**a**) Volcano plot showing differential gene expression [log2Fold change (FC)] in the neutrophil cluster from scRNAseq analysis of cells from the SRS of 15 month old *Cryba1*-floxed and cKO mice. (**b**) Ligand (L): receptor (R) pairs analysis showing expression levels (average expression and detection rate) of the indicated ligands (in microglia) and receptors (in neutrophils) in *Cryba1* cKO and floxed mice. (**c**) Ligand: Receptor pairs analysis showing scores for individual interactions in *Cryba1* cKO and floxed mice at 15 months age. Top 50 RPE-microglia interactive partners are shown with CD14-Itgb1 (integrin β1) and CD14-Itga4 (integrin α4) interactions highlighted in red, since integrin β1 and integrin α4 have been previously shown to be important in neutrophil adhesion, migration and activation. n = 3. (**d**) Schematic showing experimental design; RPE spent medium (RPESM) harvested from RPE explant cultures (24 h culture) from aged WT, *Cryba1* KO and *Akt2* KI mice was added to culture media for mouse microglia for 24 h. Then, neutrophils were co-cultured with the RPESM-exposed microglia with or without CD14 nAb (1 µg/mL) for 4 h. (**e**,**f**) Flow cytometric fluorescence plot and (**g**,**h**) graphs showing increased expression of integrin α4 (**e**,**g**) and integrin β1 (**f**,**h**) in the neutrophils co-cultured with microglia which were exposed to RPESM from *Cryba1* KO and *Akt2* KI RPE explant cultures, compared to neutrophils co-cultured with WT RPESM-exposed microglia. Treatment with CD14 nAb reduced the expression of both integrins on neutrophils (**e**–**h**), even after co-culture with activated microglia (*Cryba1* KO and Akt2 KI RPESM exposed cells). n = 4. * *p* < 0.05, ** *p* < 0.01.

Activated microglia are known to induce retinal degeneration in AMD as well as other neurodegenerative diseases [2,3,11,12,15,18,34]. We speculate that these activated microglia further exacerbate retinal inflammation in the mouse models by secreting pro-inflammatory cytokines/chemokines that would activate other innate immune cells, such as neutrophils. It is known that peripheral immune cells such as neutrophils infiltrate the SRS of a mouse model as well as in human AMD donor retina and play a critical role in the para to chronic inflammatory transition during AMD progression [16,17]. Microglia–neutrophil interaction has previously been shown to be important in other age-related diseases such as Alzheimer’s disease [36]. Currently, the extent that microglia activate neutrophils or how these immune cells interact with each other during AMD progression in the SRS remains unclear. To understand the dynamic interactions between these immune cells, we used LR loop bioinformatic analysis [25], which showed several interactive partners linking microglia and neutrophils and several differentially expressed microglial (Figure 3c) and neutrophil (Figure 5a) genes. Interestingly, we found that both CD14 (microglia)/integrin β1 (Itgβ1) (neutrophil) and CD14/integrin α4 (Itgα4) interactions were upregulated in *Cryba1* cKO retina (Figure 5a,b). CD14 is a pro-inflammatory molecule that is associated with the M1 phenotype in microglia and macrophages [37,38]. CD14 is also upregulated in microglia in neurodegenerative processes [37]. Moreover, it has been previously shown that CD14 is a ligand for integrins [39] and Itgβ1 as well as Itgα4 is known to be associated with increased neutrophil adhesion and transmigration into tissues [17,40]. Further, we have previously shown that Itgβ1 is essential for neutrophil homing into the retina [17]. Here, we tried to ascertain if RPE-derived pro-inflammatory factors can induce CD14 expression on microglia. To address this question, we cultured mouse microglia in RPESM harvested from WT, *Cryba1* KO and *Akt2* KI RPE explant cultures for 24 h and using flow cytometry and found a significantly increased CD14 levels in the *Cryba1* KO and *Akt2 KI* RPESM-exposed microglia; interestingly, these increases were rescued upon Akt2 inhibitor treatment (Supplementary Figure S3a–c). These results suggest that pro-inflammatory molecules released by the RPE could activate microglial CD14 expression through Akt2 signaling.

Further, to assess the role of microglial CD14 in regulating neutrophil adhesion molecules, mouse bone marrow-derived neutrophils were co-cultured with the RPESM-exposed microglia from all genotypes with or without CD14 neutralizing antibody (nAb) for 4 h (Figure 5d). Using flow cytometry, we found significantly elevated levels of integrins α4 (PE-A fluorescence) and β1 (FITC-A fluorescence) in neutrophils co-cultured with *Cryba1* KO and *Akt2* KI RPESM-exposed microglia (Figure 5e–h and Figure S4a–c). This effect was prevented by CD14 nAb treatment (Figure 5e–h). Moreover, no significant difference in integrins α4 and β1 expression was observed between untreated (control) neutrophils and neutrophils co-cultured with WT RPESM-exposed microglia (Supplementary Figure S4d–g). These results clearly indicate that the RPE-mediated activation of pro-inflammatory mediators on the microglial cell surface regulates the expression of neutrophil integrins, which are key to their adhesion and tissue infiltration into tissue.

### 3.4. Activated Microglia Drive Neutrophil Activation and Subsequent Retinal Degeneration

**Figure 6:**
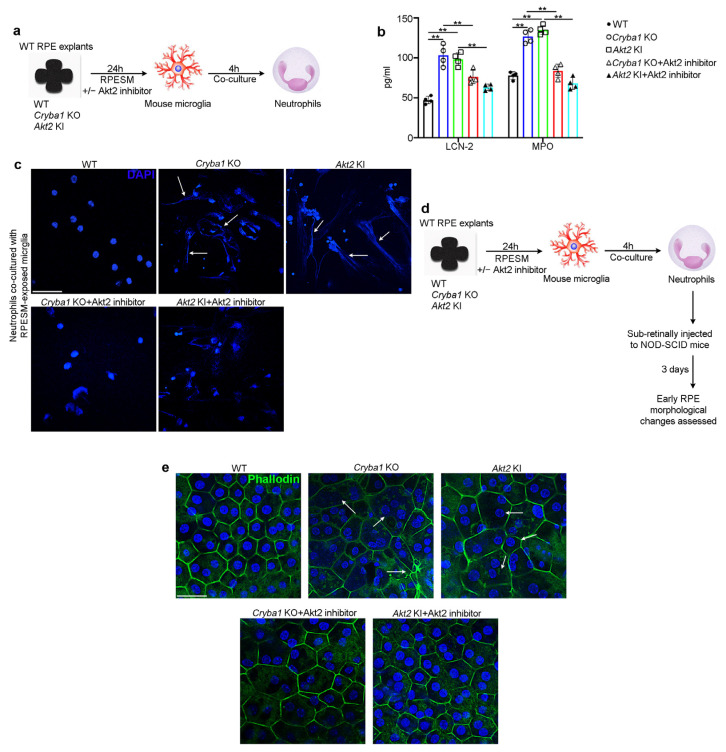
Pro-inflammatory microglial cells trigger neutrophil activation and thereby induce RPE changes. (**a**) Schematic showing experimental design; RPE spent medium (RPESM) harvested from RPE explant cultures (24 h culture) from aged WT, *Cryba1* KO and *Akt2* KI mice were used to culture mouse microglia for 24 h. Neutrophils were then co-cultured with the RPESM-exposed microglia with or without 5 nM Akt2 inhibitor. (**b**) ELISA showing significant increase in neutrophil activation markers LCN-2 and MPO in neutrophils co-cultured with microglia cells which were exposed to RPESM from *Cryba1* KO or *Akt2* KI RPE explant cultures, relative to neutrophils co-cultured with WT RPESM-exposed microglia. Akt2 inhibitor treatment to *Cryba1* KO and *Akt2* KI RPESM treated microglia before neutrophil co-culture, reduced the levels of LCN-2 and MPO in these cells. n = 4. (**c**) DAPI (blue) staining showing extended nuclear projections (NET formation) in neutrophils co-cultured with *Cryba1* KO or *Akt2* KI RPESM (arrows); Akt2 inhibitor treatment to the microglia prior to co-culturing with neutrophils markedly reduced this effect. n = 3. Scale bar = 50 µm. (**d**) Schematic showing study design for subretinal injection of neutrophils into NOD-SCID mice (as described in (**a**)). After 3 days the extent of early RPE changes was assessed. (**e**) Immunofluorescence studies on RPE flatmounts from NOD- SCID mice showing significant alterations of normal honeycomb such as morphology and enlarged cells (arrows in (**e**)) in the central region of the flatmounts from eyes injected with activated neutrophils (co-cultured with microglia which were treated with *Akt2* KI or *Cryba1* KO RPESM), compared to control. Akt2 inhibition caused marked reduction in the morphological alterations in the RPE (**e**). n = 5. Scale bar = 50 µm. ** *p* < 0.01.

In addition to adhesion and transmigration due to elevated expression of cell surface integrins, activated neutrophils express high levels of lipocalin-2 (LCN-2), myeloperoxidase (MPO), and activated neutrophil extracellular traps (NETs), which have been shown to be critical in AMD pathogenesis [17]. To pinpoint if activated (M1) microglia could induce neutrophil activation, we co-cultured mouse bone marrow-derived neutrophils with microglia pre-exposed (for 24 h) to RPESM from aged WT, *Cryba1* KO, and *Akt2* KI RPE explants. After 4 h culture (Figure 6a), we evaluated the levels of LCN-2 and MPO. Our results showed that both LCN-2 and MPO levels were significantly upregulated in neutrophils co-cultured with microglia exposed to *Cryba1* KO and *Akt2* KI RPESM, compared to WT RPESM-treated microglia (Figure 6b). Interestingly, upon Akt2 inhibitor treatment, LCN-2 and MPO levels showed noticeable decline, even after co-culture with microglia exposed to either *Cryba1* KO or *Akt2* KI RPESM (Figure 6b). We next assessed the degree of neutrophil extracellular traps (NETs) formation. NET formation is a phenotypic change typically observed in activated neutrophils which is characterized by extended nuclear processes (stained with DAPI) [41] and has been documented to be critical for degenerative processes both in AMD and Alzheimer’s disease [17,42]. Our results showed that compared to WT RPESM-treated microglia, neutrophils co-cultured with microglia which were exposed to *Cryba1* KO and *Akt2* KI RPESM showed extensive NET formation (Figure 6c), which could also be rescued upon Akt2 inhibitor treatment (Figure 6c), further establishing that M1 microglia can activate neutrophils. Given that we have previously shown that NOD-SCID (immune compromised) mice exhibit extensive retinal degenerative changes seven days after sub-retinal injection of activated neutrophils [17], we asked whether activated neutrophils induced early RPE morphological changes in NOD-SCID mice through Akt2 signaling. We injected (sub-retinally) neutrophils [17] that had been co-cultured with WT, *Cryba1* KO, or *Akt2* KI RPESM-exposed microglia, with or without Akt2 inhibitor treatment, into the sub-retinal space of NOD-SCID mice (Figure 6d). After three days we evaluated the extent of RPE morphological changes by phalloidin staining (Figure 6d). Our results showed that mice injected with neutrophils co-cultured with *Cryba1* KO and *Akt2* KI RPESM-exposed microglia triggered obvious morphological abnormalities in the central region of the RPE flatmount as evident from disorganization of honeycomb-like morphology and enlargement of cell size, as compared to RPE flatmounts from mice injected with neutrophils co-cultured with WT RPESM exposed microglia (Figure 6e). Interestingly, when the microglia were treated with Akt2 inhibitor prior to co-culture, the neutrophil injection did not produce extensive alterations in the RPE morphology (Figure 6e), likely because Akt2 inhibition also reduced microglial activation (Figure 4d,e) and subsequent neutrophil activation (Figure 6b,c). These results suggest that targeting Akt2 to reduce microglia-mediated inflammation and subsequent neutrophil activation can mitigate RPE degeneration during AMD progression.

## 4. Discussion

In AMD, inflammation is now thought to be a key factor in disease progression [2,3,4]. Intensified para-inflammation results in chronic inflammation that drives the disease process and ultimately retinal degeneration [8,9,10,11]. In animal models and human AMD patients, microglia and monocytes were found to be the dominant cells associated with disease progression [2,3,11,12,13,14,15]. Seminal previously published research has demonstrated that both microglia and monocytes infiltrate the SRS in the disease state [11,12,13,14,15]. It is known that the retina has two distinct microglia pools differing by niche and IL-34 dependency [12]. In the normal retina, IL-34-dependent microglia contribute to neuronal function, whereas, during degeneration, these populations of microglia move toward the RPE cells into the SRS, an inducible disease-associated niche [12]. This microglial transition results from extensive transcriptional reprogramming of microglia, characterized by reduced expression of homeostatic checkpoint genes and upregulation of injury-responsive genes [12]. Genetic polymorphisms associated with AMD have been linked with the activation of microglia and monocytes, their infiltration into the SRS, and the subsequent induction of pathogenic chronic inflammation [26,27]. As an example, genetic variants of *Cx3cr1* (a microglial homeostatic marker gene that is not expressed by any other cells in the retina), apolipoprotein E2 isoform (APOE), and complement factor H (Y402H) are associated with increased accumulation of microglia and monocytes in the SRS in human AMD patients [11,12,13,14,15]. In animal models, this accumulation of immune cells has been shown to trigger photoreceptor degeneration [11,12,13,14,15,16,17]. Moreover, in mice loss of Cx*3cr1* induces a pro-inflammatory activation of microglia and triggers sub-retinal accumulation of monocytes [11,12,15]. Importantly, the age-dependent accumulation of subretinal monocytes in *Cx3cr1*-deficient mice is associated with a significant degeneration of rods and cones [11,43].

Microglia and monocytes are now established as major immunomodulators in AMD [11], but it is hard to believe that these immune cells are the only ones responsible for disease progression in a multi-factorial disease such as AMD. Currently, the specific roles of other immune cells, particularly neutrophils, remain uncertain in AMD pathogenesis. It has previously been shown that the number of neutrophils in the peripheral blood of neovascular (wet) AMD patients is highly elevated, and that the resolution of the inflammation marker (CXCR2) on the neutrophils is downregulated [44]. In addition, we have shown that neutrophils infiltrate the retina in human dry/atrophic AMD patients [16,17] and in a mouse model exhibiting a slow progressive atrophic (early/dry) AMD-like pathology [17]. This neutrophil infiltration correlates with development of an aging-related chronic inflammatory response [16,17]. Our studies revealed that activation of Akt2 signaling in the RPE triggers neutrophil infiltration in human AMD patients and in our mouse model, and that this infiltration is associated with retinal degeneration [17], a finding confirmed by the fact that inhibition of Akt2 in the mouse model reduced neutrophil infiltration and alleviated early RPE changes [17].

The infiltration of microglia and neutrophils into the SRS during AMD progression is well documented [11,12,17] but a major gap in our understanding is how these immune cells interact with each other in the diseased state. It is known that under normal physiological conditions, the homing of neutrophils into the retina is restricted as they are effectively eliminated by microglia, thereby maintaining tissue homeostasis [10,11]. However, the nature of their interactions in the retina during AMD progression is unknown. In other diseases such as Alzheimer’s disease, stroke, and intracerebral hemorrhage, it has been previously documented that activated microglia facilitate neutrophil survival, activation, and migration leading to tissue damage [45,46,47]. To assess the role of microglia/neutrophil interaction in AMD progression, we performed scRNASeq analysis on cells from the SRS isolated from *Cryba1* cKO mice employing a novel bioinformatic tool, LR-loop [25]. This identified several interacting partners linking the two cell types, suggesting that microglia–neutrophil interaction is an important factor in the AMD-like pathology in this mouse model. It is likely that the chronic inflammatory transition in AMD pathogenesis results from alterations in these microglia–neutrophils interactions, where protective microglia would normally eliminate any infiltrating neutrophils during the normal aging process [36]. However, as a consequence of genetic predisposition or lifestyle/environmental factors microglia can transition into an M1 (pro-inflammatory state) which in turn could activate other immune cells and lead to retinal degeneration [8,9,10,11].

**Figure 7:**
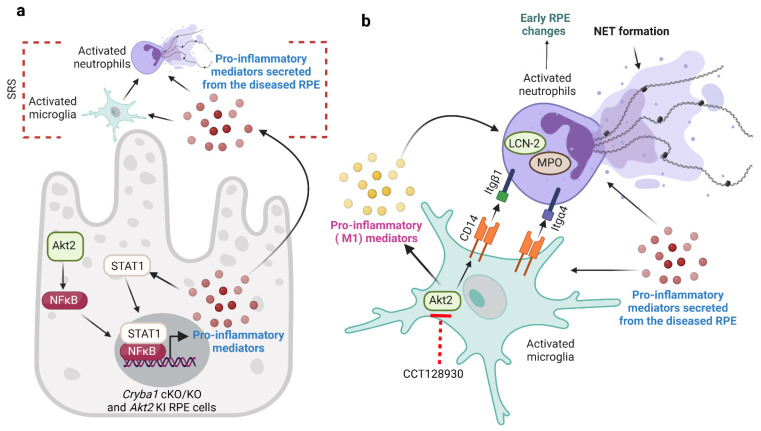
Microglia–neutrophil interaction in AMD pathogenesis. (**a**) RPE cells from aged *Cryba1* cKO and *Akt2* KI mice secrete pro-inflammatory mediators (cytokines and chemokines) due to activation of AKT serine/threonine kinase 2 (Akt2)/nuclear factor kappa-light-chain-enhancer of activated B cells (NFκB) signaling. These pro-inflammatory mediators trigger microglia and neutrophils infiltration into the subretinal space (SRS). (**b**) Cytokines and chemokines secreted by the RPE cells induce pro-inflammatory (M1) transition in microglia through Akt2 activation. These activated microglia release pro-inflammatory mediators and express high levels of cluster of differentiation 14 (CD14) on the cell surface, which in turn activates neutrophils as evident from elevated lipocalin-2 (LCN-2), myeloperoxidase (MPO), and neutrophil extracellular trap (NET) formation, thereby inducing early retinal pigmented epithelium (RPE) changes in nonobese diabetic/severe combined immunodeficiency (NOD-SCID) mice. Further, the M1 microglia can regulate the expression of adhesion proteins such as integrin beta 1 (Itgβ1) and integrin alpha 4 (Itgα4) on neutrophils through their interactions with the high levels of CD14 on the surface of microglia. Interestingly, inhibiting Akt2 in microglia reduced the release of pro-inflammatory cytokines and reduced CD14 levels in these cells, diminishing neutrophil activation in vitro and subsequent changes in morphology in RPE morphology in vivo. Created with BioRender.com (accessed on 30 August 2022).

In this study, we provide novel evidence regarding the role of microglia/neutrophil interaction in AMD pathogenesis. We observed that RPE cells drive the transition of microglia into a pro-inflammatory (M1) phenotype leading to their migration into the SRS in our mouse models. It has been previously shown that RPE-derived inflammatory factors trigger immune cell activation [11,12,13,14,15,16,17]. We show that the process of microglia activation is dependent on Akt2 signaling and that inhibiting Akt2 in microglia reduced the levels of secreted pro-inflammatory mediators (Figure 7). Akt2 has been shown to be indispensable for M1 transition in macrophage-lineage cells [29,33,48]. Macrophages from Akt2 knockout mice remain in an anti-inflammatory (M2) phenotype even after exposure to LPS, a known activator of the M1 phenotype [48]. Targeting Akt2 in activated microglia using gene silencing approaches has also been previously shown to curb the M1 phenotype and confer protection in animal models of demyelination [33]. We also show that M1 microglia trigger neutrophil activation (elevated LCN-2, MPO and NET formation) that subsequently induces early RPE morphological changes in NOD-SCID mice. Further, our study shows that CD14 on M1 microglial cell surface can regulate the expression of neutrophil adhesion proteins such as integrin β1 and α4 (Figure 7), which have been previously shown to be important for neutrophil migration and activation [17,40]. CD14 is an important ligand for integrins and shows an activation state-dependent binding thereby maintaining integrin expression on the cell surface [39]. Elevated levels of CD14 have been associated with other neurodegenerative diseases such as Alzheimer’s disease [37]. Interestingly, inhibiting CD14 and microglial activation in vitro via Akt2 inhibition resulted in rescue of neutrophil activation and reduction of cell surface adhesion protein levels (Figure 7). These results strongly suggest that microglia–neutrophil interaction is important in the pathogenesis of AMD. Future studies should aim to elucidate the nature of these intercellular processes as a means of identifying new targets and approaches for treating or preventing dry AMD.

## Data Availability

All data generated or analyzed during this study are included in this published article (in the main figures and the Supplementary Figures). The raw uncropped Western blot images are supplied in a single file. The scRNAseq data will be uploaded in the NCBI GEO database immediately following acceptance. The schematic (Figure 7) is original and was created at BioRender.com (accessed on 30 August 2022).

## Supplementary Materials

The following supporting information can be downloaded at: https://www.mdpi.com/article/10.3390/cells11223535/s1, Supplementary Figures S1–S4 and the raw uncropped Western blot images are included as separate files.
