# Supplementary material for: Microglia–Neutrophil Interactions Drive Dry AMD-like Pathology in a Mouse Model
Source: Cells. 2022 Nov 9;11(22):3535. doi: 10.3390/cells11223535 (PMC9688699; doi:10.3390/cells11223535)
Supplement: Supplementary file 1 [file cells-11-03535-s001.zip › cells-1924211-Supplementary Figures Legends latest.pdf]

Supplementary Figure S1

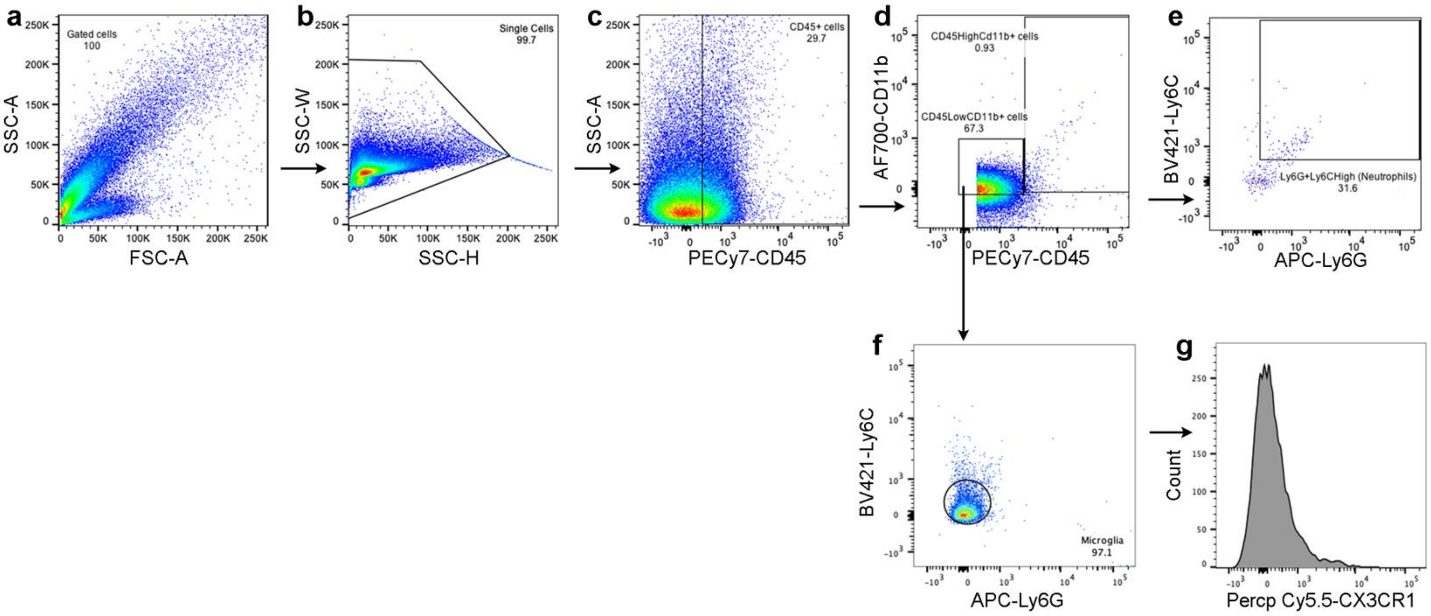

<u>Figure S1.</u> Gating strategy for immunophenotyping of cells from the subretinal region. (**a-f**) Representative dot plots showing gating strategy for $CD45^{+}CD11b^{+}$ cells from SRS. The $CD45^{high}CD11b^{+}$ and $CD45^{low}CD11b^{+}$ were gated separately (arrows denoting population lineages). The levels of Ly6C and Ly6G on the $CD45^{high}CD11b^{+}$ population were assessed to evaluate the percentage of neutrophils ($CD45^{high}CD11b^{+}Ly6C^{high}Ly6G^{+}$ cells) and microglia ($CD45^{low}CD11b^{+}Ly6C^{-}Ly6G^{-}$ cells). The expression of CX3CR1 was evaluated among the microglia population (**g**).

Supplementary Figure 2

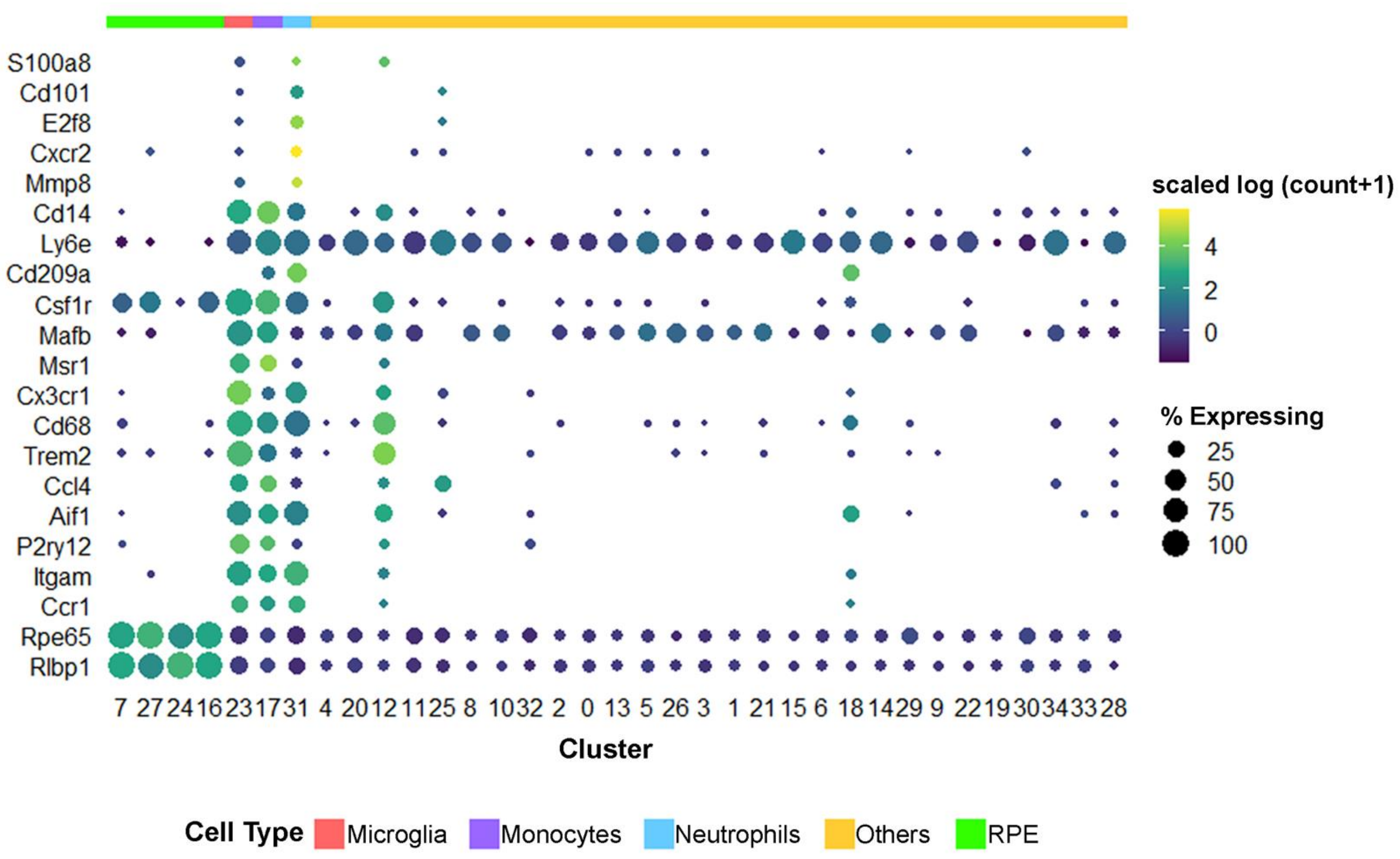

Figure S2. Cell type identification from scRNAseq analysis. Dot plot showing expression profiles for specific marker genes for RPE (Green), monocytes (Purple), neutrophils (Cyan) and microglia (Red) in the 35 clusters identified from scRNAseq data of the sub-retinal region from *Cryba1* cKO mice (3 and 15 month data integrated). N = 3.

Supplementary Figure 3

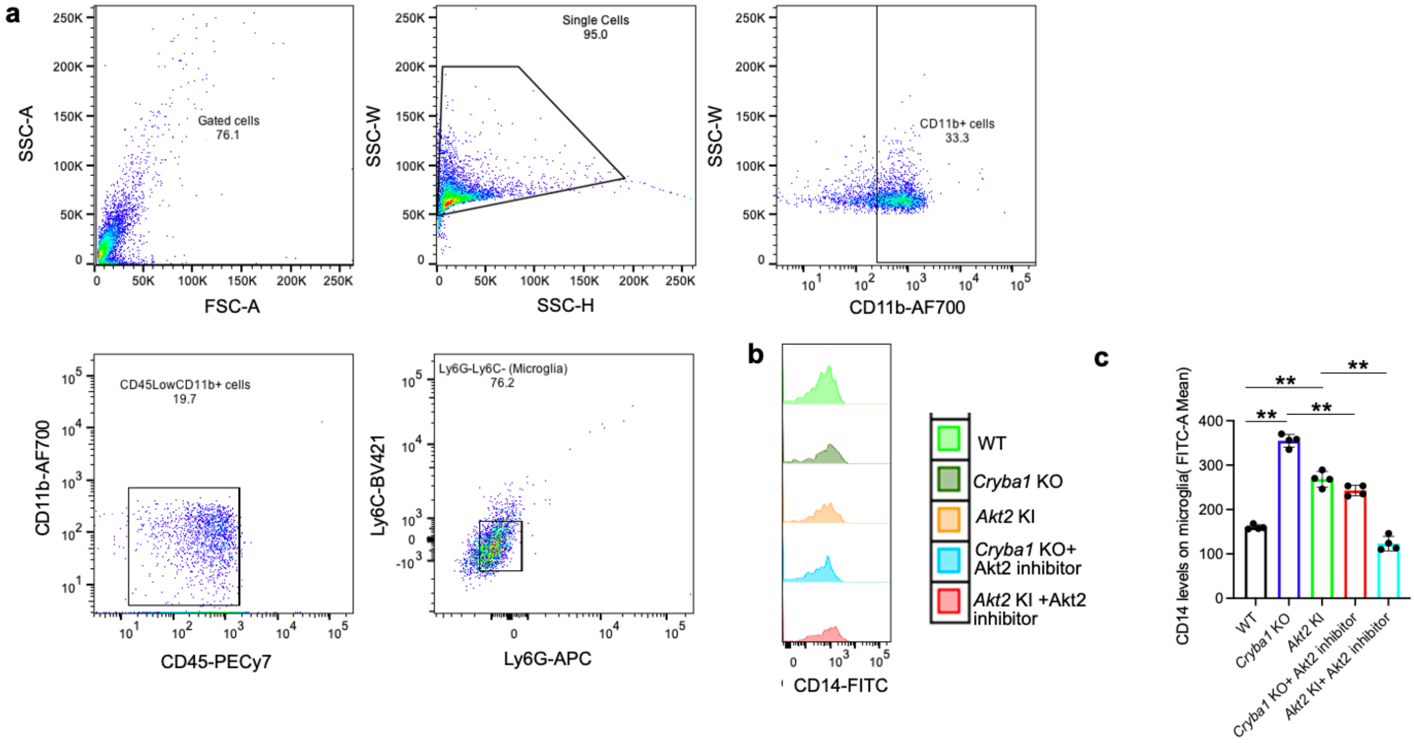

Figure S3. Akt2 inhibition downregulates CD14 expression on microglia. (**a**) Representative dot plots showing the gating strategy for $CD45^{+}CD11b^{+}$ cells from mouse microglia culture. The $CD45^{low}CD11b^{+}$ were gated and the level of Ly6C and Ly6G among $CD45^{low}CD11b^{+}$ cell population was assessed to identify microglia ($CD45^{Low}CD11b^{+}Ly6C^{-}Ly6G^{-}$). The levels of CD14 (FITC-A Mean) were evaluated. (**b**) Flow cytometric fluorescence plot and (**c**) graph showing increased expression of CD14 (FITC-A Mean) in microglia treated with RPESM from *Cryba1* KO or *Akt2* KI mice compared to WT RPESM treated cells, which was rescued upon Akt2 inhibition in the microglial cells. n = 4. * $P < 0.05$, ** $P < 0.01$.

Supplementary Figure 4

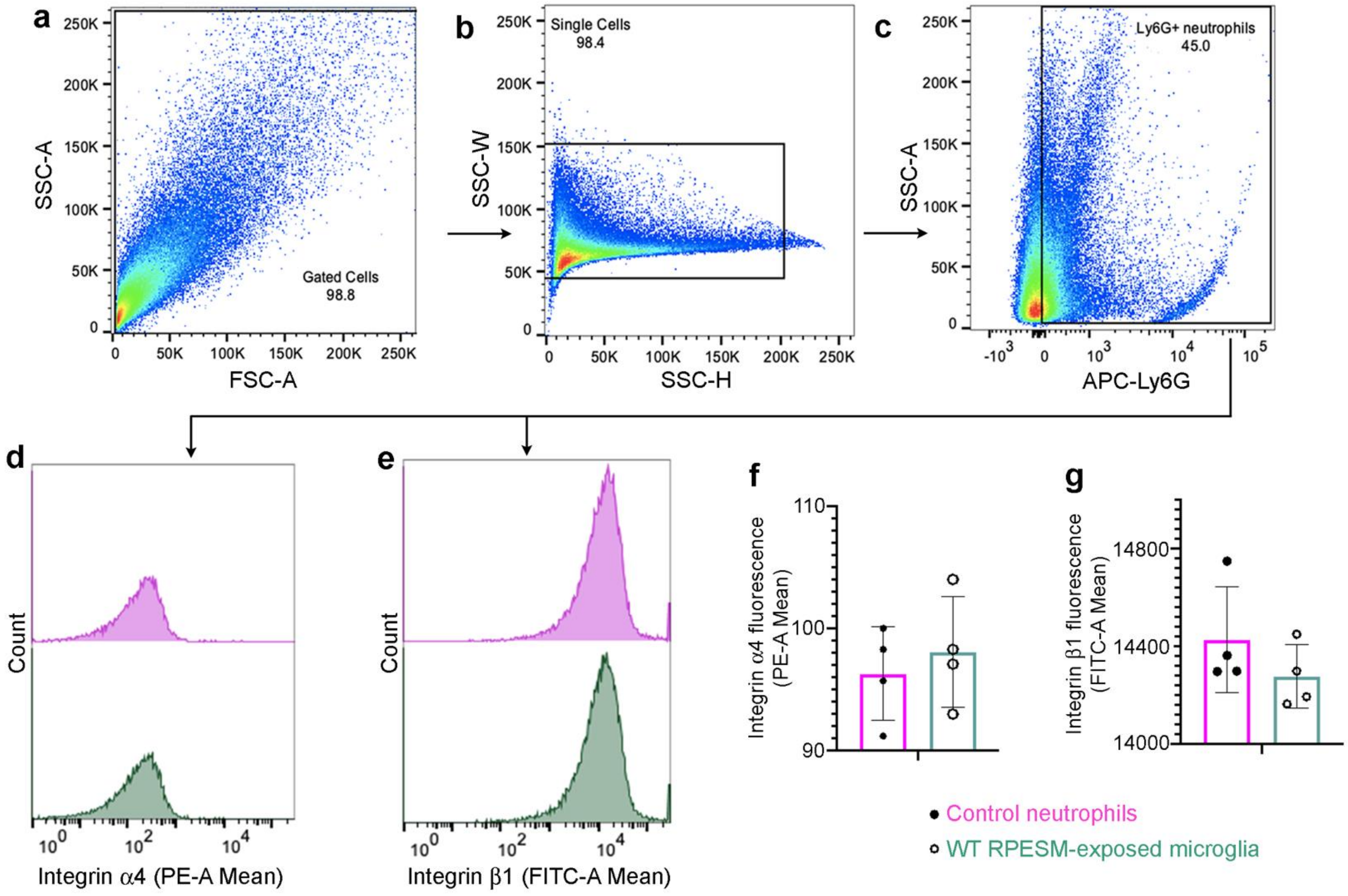

Figure S4. Integrins α4 and β1 expression on neutrophils. (**a-c**) Representative dot plots showing the gating strategy for cultured neutrophils +/− co-culturing with microglia. (**d-g**) The integrin α4 (PE-A Mean) and β1 (FITC-A Mean) levels were evaluated among Ly6G+ cells in untreated (control) and in neutrophils co-cultured with WT RPESM-exposed microglia, which showed no significant difference. n = 4.
