# Supplementary figures and images for: Microglia–Neutrophil Interactions Drive Dry AMD-like Pathology in a Mouse Model
Source: Cells. 2022 Nov 9;11(22):3535. doi: 10.3390/cells11223535 (PMC9688699; doi:10.3390/cells11223535)

**Supplementary Information: Figure 4b uncropped blot**

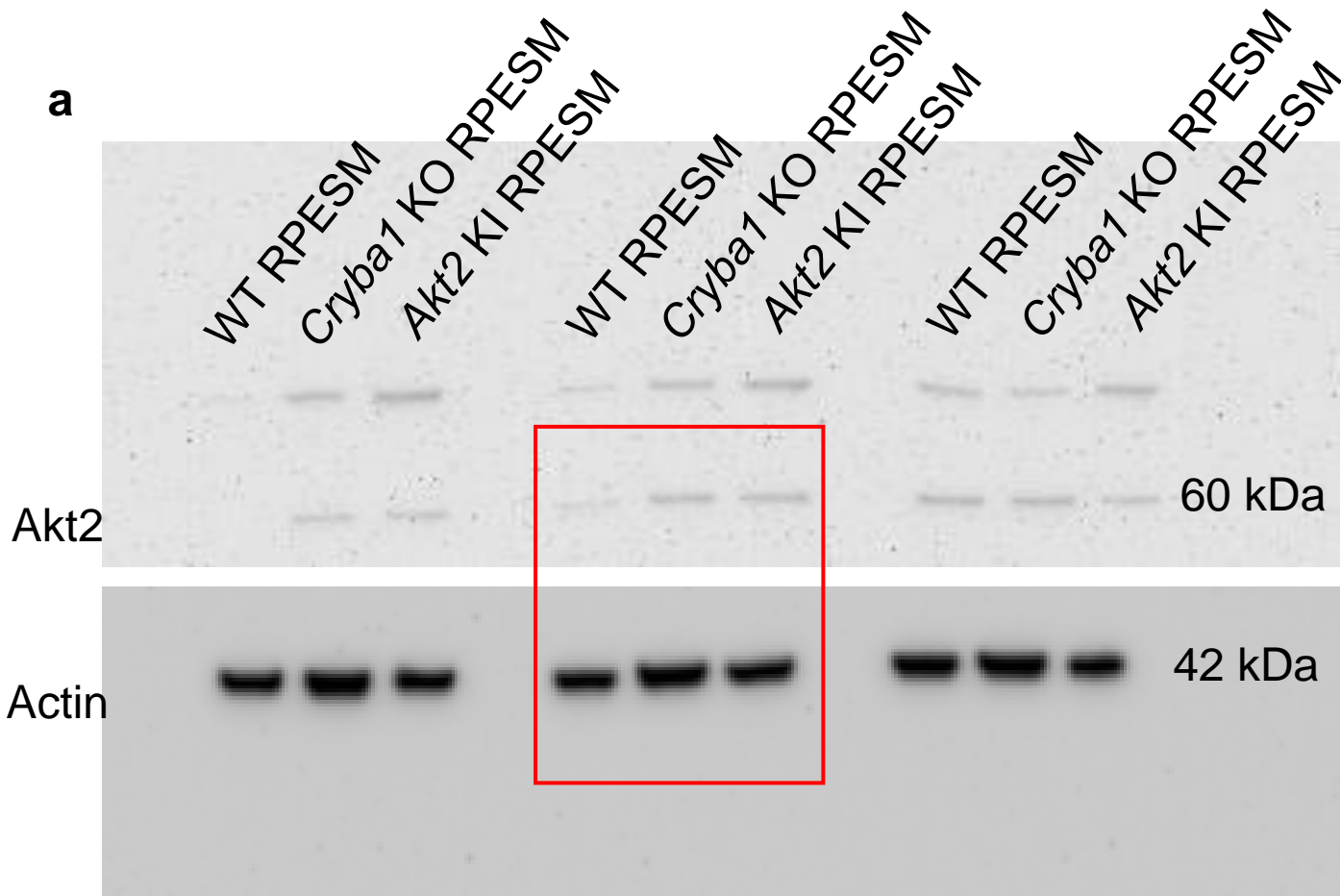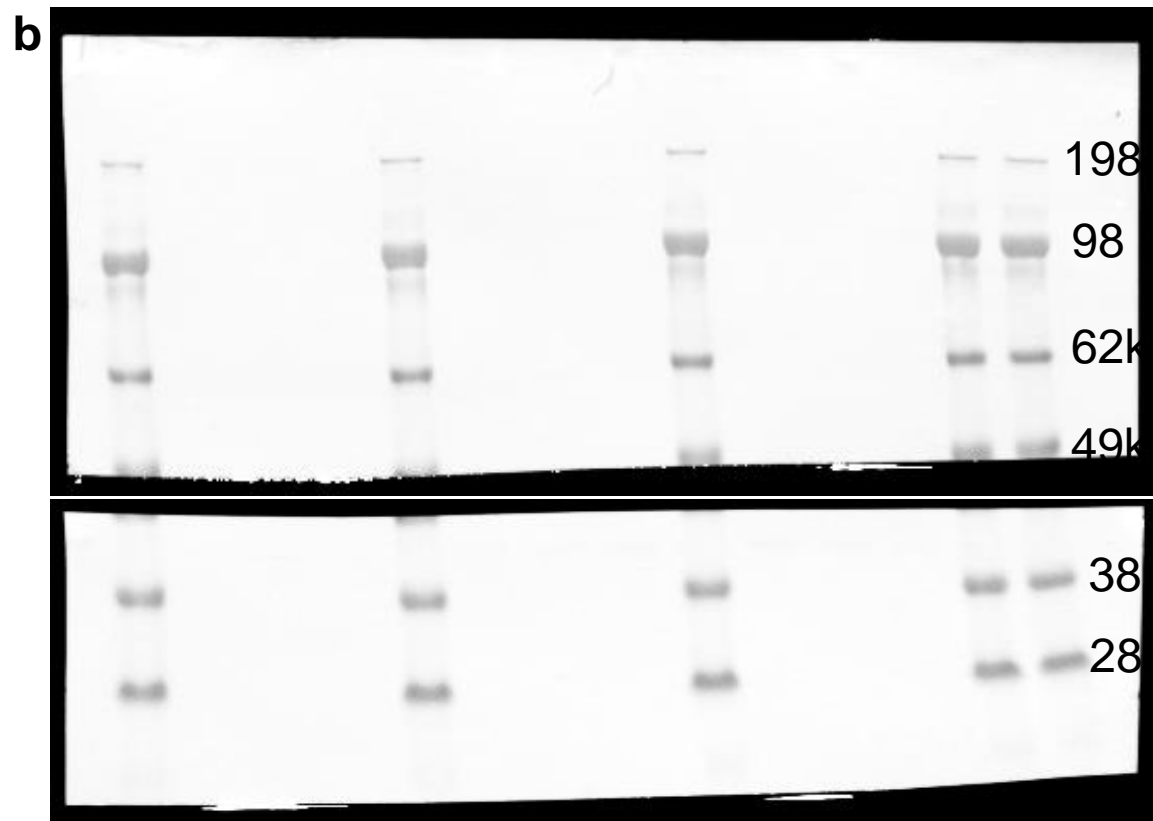

Supplement: Supplementary file 1 [file cells-11-03535-s001.zip › cells-1924211-Supplementary Information.pdf]
